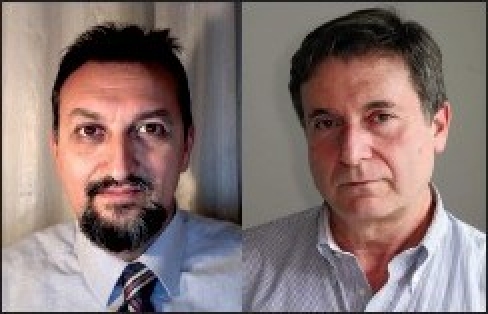# Calorimetric techniques to study the interaction of drugs with biomembrane models

**DOI:** 10.4103/0975-7406.76459

**Published:** 2011

**Authors:** Rosario Pignatello, Francesco Castelli

**Affiliations:** Professor of Pharmaceutical Technology and Legislation, University of Catania, Italy; 1 Professor of Applied Pharmaceutical Chemistry, University of Catania, Italy E-mail: r.pignatello@unict.it,fcastelli@unict.it,editor@jpbsonline.org

Design and development of drug compounds and new pharmaceutical formulations require full characterization of the chemical and physicochemical events occurring at the level of the single active ingredients or excipients, as well as following their reciprocal interaction. Thermal analysis techniques are among the most widely used methods to this aim; among them, by using the Differential Scanning Calorimetry (DSC) technique, the thermotropic behavior of a single substance or mixtures is analyzed as a function of a controlled temperature program.

Differential Scanning Calorimetry is an accurate and rapid thermoanalytical technique, widely used by the pharmaceutical industry and in drug research, to investigate several physicochemical phenomena, such as polymorphism, melting and crystallization, purity, drugexcipient interaction, polymer properties, effects of drying and lyophilization, as well as to characterize biomolecules like peptides, proteins, and genetic material.

A recent interrogation of the Pubmed database (December 2010) on publications containing the words ‘Differential Scanning Calorimetry’ (DSC) gave a total number of about 12,900 items. Out of them, a cross analysis between DSC and the terms ‘drug*’ or ‘pharm*’ reduced the number to about 4,250 and 2,800 items, respectively. A search for ‘DSC’ and ‘membrane’ gave about 2,120 publications, 130 of which were also related to ‘biomembranes’. These figures very well corroborate the value and significance of such an analytical technique in the development and optimization of new drug compounds and their final formulations.

However, peculiar applications of DSC in biomedical research are also possible, and according to us, not all of them have been explored yet. For instance, DSC can be a very powerful tool and the source of a large amount of information, to study the interactions between drugs and cell membranes. This can be due to both the qualitative and quantitative levels, and using either eukaryotic or bacterial cell membranes, as well as different biomembrane models. In fact, the 3-D nature of these systems allows to put into evidence the different possible mechanisms and degrees of interactions between a biologically active molecule and biomembranes, much better than 2-D (i.e., solvent-solvent) experimental approaches. The database returned only 21 hits when the terms ‘DSC’ and ‘(bio)membrane model*’ were put in, and only 14 when the terms ‘drug’, ‘membrane interaction,’ and ‘DSC’ were cross-queried. This meant that a large amount of work could still be done, to make DSC a routine technique in this specific phase of drug design and development.

In this special issue we have invited some scientists renowned for their work in the field of DSC applications to drug development and delivery, and especially to drugbiomembrane interaction studies, to contribute in clarifying some of the above-mentioned issues and to highlight areas where uncertainties remain. A fundamental challenge is to combine insights from biochemistry and physiology with those from structural biology and bio-thermodynamics, to obtain a complete depiction of cell membranes and their functions. The review of Prof. Raudino (Department of Chemical Sciences, University of Catania, Italy) brilliantly delineates the physical principles and the thermodynamic techniques staying at the basis of the biological membrane models. The great number of experimental data must be interpreted on the basis of approximate, but not over-simplified, models; Prof. Pignatello (Department of Drug Sciences, University of Catania, Italy) has critically analyzed the literature regarding the *in vitro* biomembrane models and their significance in the development of new drugs and medicines. Prof. Chiu and Prof. Prenner (Department of Biological Sciences, University of Calgary, Canada) have instead reviewed the applications of DSC in biochemical and pharmaceutical studies and highlighted the numerous shortcomings of the approaches used and the results reported in recent literature.

An interesting and particular application of DSC for studying the release rate and kinetics of drugs from colloidal nanovectors to biomembrane models has been reviewed by Prof. Sarpietro and Prof. Castelli (Department of Drug Sciences, University of Catania, Italy), whereas Prof. Bastos (Department of Chemistry and Biochemistry, University of Porto, Portugal) has contributed with a research illustrating the application of DSC in characterizing the structure-activity profile of peptide antimicrobials derived from lactoferrin. Finally, Prof. Giatrellis (Department of Medical Biochemistry and Biophysics, Umeå University, Sweden) and Prof. Nounensis (Biomolecular Physics Laboratory, NCSR Demokritos, Greece) have made a critical analysis of the literature on DSC studies on nucleic acid-membrane systems, discussing the experimental data related to the thermodynamics and kinetics of DNA-lipid complexation, and especially to the lipid organization and phase transitions within the membrane model.

We are grateful to all the co-authors for the production of the above-mentioned articles and reviews; we are also indebted to the scientists who have peer-reviewed the manuscripts and given valuable advice to the authors and ourselves, whereby all had to schedule their research around a tight time scale. We are also extremely grateful to the Editor-in-Chief, Professor R. K. Khar, Dr. M. Aqil, Editor of the Pharmaceutical Sciences section, Dr. H. Gupta, Head of the Management Board, and the OPUBS group, to have accepted the proposal of this special issue.